# Nocapyrones: α- and γ-Pyrones from a Marine-Derived *Nocardiopsis* sp.

**DOI:** 10.3390/md12074110

**Published:** 2014-07-08

**Authors:** Youngju Kim, Hiromu Ogura, Kazuaki Akasaka, Tsutomu Oikawa, Nobuyasu Matsuura, Chiaki Imada, Hisato Yasuda, Yasuhiro Igarashi

**Affiliations:** 1 Biotechnology Research Center and Department of Biotechnology, Toyama Prefectural University, 5180 Kurokawa, Imizu, Toyama 939-0398, Japan; E-Mails: youngju.kim.c@gmail.com (Y.K.); qqct6qc9k@blue.ocn.ne.jp (H.O.); 2 Shokei Gakuin University, 4-10-1 Yurigaoka, Natori, Miyagi 981-1295, Japan; E-Mail: akasaka@shokei.ac.jp; 3 School of Nutrition and Dietetics, Kanagawa University of Human Services, 1-10-1 Heisei-cho, Yokosuka, Kanagawa 238-8566, Japan; E-Mail: oikawa-t@kuhs.ac.jp; 4 Okayama University of Science, 1-1 Ridai-cho, Okayama, Okayama 700-0005, Japan; E-Mail: nobuyasu@dls.ous.ac.jp; 5 Tokyo University of Marine Science and Technology, 4-5-7 Konan, Minato-ku, Tokyo 108-8477, Japan; E-Mail: imada@kaiyodai.ac.jp; 6 Center for Advanced Marine Core Research, Kochi University, B200 Monobe, Nankoku, Kochi 783-8502, Japan; E-Mail: yasuda@kochi-u.ac.jp

**Keywords:** adiponectin, adipocyte, α-pyrone, γ-pyrone, *Nocardiopsis*

## Abstract

One new α-pyrone (nocapyrone R (**1**)), and three known γ-pyrones (nocapyrones B, H and L (**2**–**4**)) were isolated from the culture extract of a *Nocardiopsis* strain collected from marine sediment. Structures of these compounds were determined on the basis of spectroscopic data including NMR and MS. γ-Pyrones **2**–**4** were found to induce adiponectin production in murine ST-13 preadipocyte cells but the α-pyrone **1** had no activity. The absolute configuration of the *anteiso*-methyl branching in **4** was determined by HPLC comparison of a degraded product of **4** with standard samples as a 2:3 enantiomeric mixture of (*R*)- and (*S*)-isomers.

## 1. Introduction

Natural products have been playing an important role for the development of novel therapeutics owing to their enormous and unpredictable structural diversity [1]. Specifically, actinomycetes are continuously providing structurally diverse secondary metabolites possessing pharmaceutically useful bioactivities. More than 70% of microbial antibiotics have been discovered from actinomycetes [2]. In recent years, in addition to the terrestrial species, actinomycetes from marine environments are attracting a substantial attention as a new resource of novel drug candidates [3]. The differences between the marine and terrestrial environments are likely reflected in the genetic divergences. Marine-derived actinomycetes are recognized to actually produce a range of chemically distinctive secondary metabolites that terrestrial actinomycetes are not able to produce [4]. Consequently, the number of novel metabolites from marine actinomycetes has been increasing in recent years.

**Figure 1:**
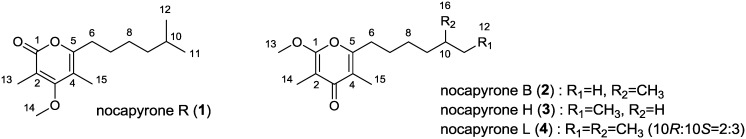
Structures of nocapyrones R (**1**), B (**2**), H (**3**), and L (**4**).

The increasing incidents of type 2 insulin-resistant diabetes, as a result of growing obesity rates, are causing a serious social and economic problem [5]. Adipose tissue secretes various types of biologically active adipokines, such as free fatty acids, tumor necrosis factor-α, adiposin, resistin, and leptin, to regulate energy homeostasis [6]. Adiponectin is one of such adipokines secreted exclusively by mature adipocytes [7] and this proteineous substance functions to regulate the glucose and lipid metabolism [8]. Recent research revealed that the mRNA expression of adiponectin is reduced in obese diabetic murine model [9] and type 2 diabetic patients [10,11,12], and also plasma level of adiponcetin is significantly lower in obese diabetic mice and humans [13,14,15]. Therefore, the replenishment of adiponectin by transcriptional induction in adipocytes is believed to provide a new effective therapeutic approach to insulin resistance, type 2 diabetes, and related diseases. Thiazolidinediones represented by rosiglitazone (Avandia) and pioglitazone (Actos) are widely-used orally available drugs for type 2 diabetes. These synthetic compounds activate transcription by PPARγ (peroxisome proliferator activated receptor γ) primarily in adipose tissues and induce the elevation of adiponectin plasma level. Meanwhile, several research groups have demonstrated that serious side effects are caused by rosiglitazone; the use of this drug is associated with a 43% increase in myocardial infraction and 64% increase in the risk of death from cardiovascular causes [16]. Thus, the development of side effect-free new agents for type 2 diabetes is desperately desired. In our screening program for discovering new lead scaffolds for adiponectin inducers from natural products [17,18,19], a marine-derived *Nocardiopsis* strain was found to produce a new α-pyrone (**1**) along with three known γ-pyrones, nocapyrones B, H, and L (**2**–**4**, Figure 1). Interestingly, nocapyrone L (**4**) was isolated as an enantiomeric mixture, which is noticeable in marine natural products. Herein, we describe isolation, structure determination, and biological activities of these compounds.

## 2. Results and Discussion

### 2.1. Structure Analysis and Characterization

#### 2.1.1. Isolation

The producing strain TP-A0876 was isolated from a sediment sample collected at −775 m in Ishikari gulf, Hokkaido, Japan. On the basis of the result of 16S rRNA gene sequence similarity, the isolate was identified as *Nocardiopsis* sp. The strain TP-A0876 was cultured in A-3M medium at 30 °C for 6 days and the whole culture broth was extracted with 1-butanol. The crude extract was subjected to silica gel and ODS column chromatographies, followed by HPLC purification, to yield nocapyrone R (**1**) along with nocapyrones B, H, and L (**2**–**4**), three known compounds previously isolated from marine *Nocardiopsis* strains by other groups [20,21].

#### 2.1.2. Nocapyrone R (**1**)

**Figure 2:**
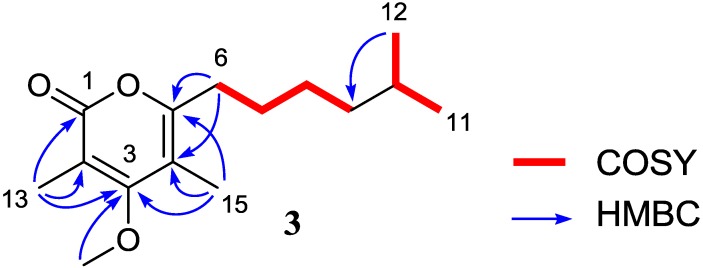
2D-NMR correlations for nocapyrone R (**1**).

Nocapyrone R (**1**) was isolated as colorless amorphous. The molecular formula of **1** was determined as C_15_H_24_O_3_ by high resolution ESITOFMS (Electrospray Ionization Time Of Flight Mass Spectrometry) analysis that showed a pseudomolecular ion at *m*/*z* 275.1619 [M + Na]^+^ (calcd for C_15_H_24_O_3_Na, 275.1623), which was corroborated by the ^1^H and ^13^C NMR data. The IR spectrum indicated the presence of alkene (2944 cm^−1^) and carbonyl (1660 cm^−1^) groups. ^1^H and ^13^C NMR data in combination with the HSQC (Heteronuclear Single Quantum Coherence) analysis revealed the presence of 15 carbons assignable to one carbonyl carbon, four sp^2^ quaternary carbons, four sp^3^ methylenes, one sp^3^ methine, and five methyl groups (one is oxygenated) (Table 1, Supplementary Figures S1, S2 and S4). The NMR spectra of nocapyrone R (**1**) showed the resonances for a pyrone unit as well as two methyl (C-13 and C-15) and one methoxy (C-14) substituents (Table 1, Supplementary Figures S1 and S2): however, the UV spectrum showing absorption maximum at λ_max_ 290 nm was different from that for γ-pyrone (**2**–**4**: UV λ_max_ 252 nm). Furthermore, the ester carbonyl absorption of IR spectrum and the ester carbonyl signal of ^13^C NMR suggested the presence of an ester/lactone functionality. Protons of two olefinic methyl groups (H_3_-13 and H_3_-15) had HMBC correlations with C-3, to which was correlated the methoxy protons (H_3_-14), establishing the α-pyrone substructure with a methoxy group at C-3 flanked by two olefinic methyl groups (Supplementary Figure S5). The ^1^H-^1^H COSY spectrum provided two fragments, H_2_-6/H_2_-7/H_2_-8/H_2_-9 and H_3_-11/H-10/H_3_-12. These two fragments were assembled into an alkyl chain bearing an isopropyl terminus by HMBC correlations from H_3_-12 to C-9, C-10, and C-11. This was consistent with the presence of two doublet methyl proton signals for H_3_-11 and H_3_-12 (Supplementary Figures S3 and S5). The alkyl side chain was attached to C-5 by HMBC correlations from H_2_-6 to C-4 and C-5, completing the structure of **1** (Figure 2).

**Table 1:** ^1^H and ^13^C NMR data for nocapyrone R (**1**) in CDCl_3_.

Position	δ_C_^a^	δ_H_ mult (*J* in Hz) ^b^	COSY ^b^	HMBC ^b,c^
1	166.3, qC			
2	109.3, qC			
3	168.4, qC			
4	109.0, qC			
5	159.3, qC			
6	31.1, CH_2_	2.49, t (7.8)	7	4, 5, 7
7	27.7, CH_2_	1.61, m	6, 8	5
8	27.1, CH_2_	1.31, m	7, 9	10
9	38.7, CH_2_	1.18, m	8	7, 11, 12
10	27.9, CH	1.52, m		11, 12
11	22.6, CH_3_	0.86, d (6.5)	10, 12	9, 10, 12
12	22.6, CH_3_	0.86, d (6.5)	10, 11	9, 10, 11
13	10.2, CH_3_	2.03, s		1, 2, 3
14	60.2, CH_3_	3.80, s		3
15	10.1, CH_3_	1.92, s		3, 4, 5

^a^ recorded at 100 MHz; ^b^ recorded at 500 MHz; ^c^ HMBC correlations are from proton(s) stated to the indicated carbon.

#### 2.1.3. Absolute Configuration of Nocapyrone L (**4**)

**Scheme 1:**
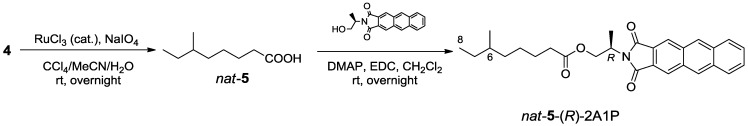
Degradation of **4** to **5** and labeling with (*R*)-2A1P.

**Figure 3:**
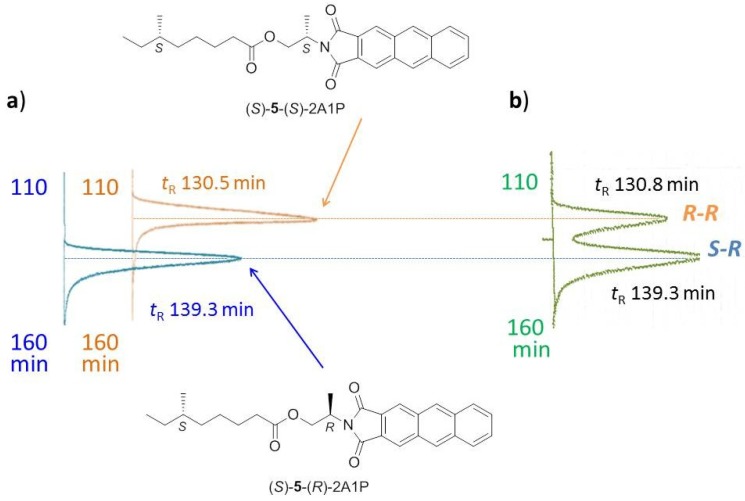
Determination of absolute configuration of *anteiso*-methyl group by HPLC. (**a**) HPLC chromatogram of standard (*S*)-**5**-(*S*)-2A1P (right graph) and (*S*)-**5**-(*R*)-2A1P (left graph); (**b**) HPLC chromatogram of *nat*-**5**-(*R*)-2A1P.

**Figure 4:**
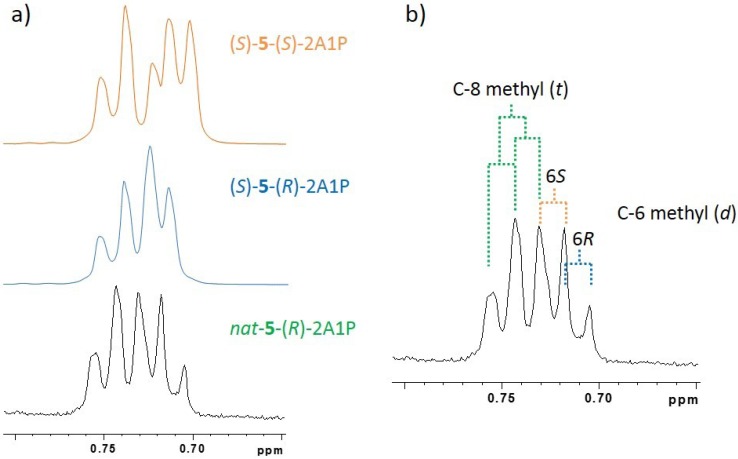
Expanded ^1^H NMR spectra of *nat*-**5**-(*R*)-2A1P, standard (*S*)-**5**-(*S*)-2A1P and (*S*)-**5**-(*R*)-2A1P. (**a**) comparison of *nat*-**5**-(*R*)-2A1P (bottom) with standard (*S*)-**5**-(*S*)-2A1P (top) and (*S*)-**5**-(*R*)-2A1P (middle); (**b**) analysis of ^1^H NMR signal of *nat*-**5**-(*R*)-2A1P.

Nocapyrone L (**4**) has been reported as a secondary metabolite of *Nocadiopsis* sp. isolated from a mollusk as a symbiotic bacterium [21]. Due to the remoteness of the branched methyl chiral center (C-10) from the modifiable functional groups, the absolute configuration of **4** had not been assigned. From the structural analogy of **4** to germicidin A, we hypothesized that **4** could be biosynthesized through condensation of an amino acid-derived starter with malonate extenders [22]. Consequently, the *anteiso*-subunit known to be originated from l-isoleucine is assumed to have *S* configuration. Interestingly, the specific rotation of **4** we isolated showed [α]_D_^25^ = −2.2 (*c* = 0.1, CHCl_3_), while that of reported one is [α]_D_^25^ = +9.0 (*c* = 0.1, CHCl_3_) [21]. In order to clarify the absolute configuration of **4**, Ohrui-Akasaka method was applied (Scheme 1) [23]. The pyrone unit of **4** was oxidatively degraded by the treatment with a catalytic amount of ruthenium (III) chloride and sodium periodate in a biphasic solvent system (CCl_4_–MeCN–H_2_O) to give 6-methyloctanoic acid **5** [24]. This *anteiso*-fatty acid was then esterified with a chiral anthracene reagent, (*R*)-2-(anthracene-2,3-dicarboximido)propanol [(*R*)-2A1P], to yield *nat*-**5**-(*R*)-2A1P (Supplementary Figure S6). HPLC analysis of this derivative was performed on Develosil ODS-HG-3 column (4.6 mm × 150 mm, column temp. −58.5 °C) with the eluent of MeOH/MeCN/THF (4:3:1) at a flow rate of 0.2 mL/min. (*S*)-6-Methyloctanoic acid [(*S*)-5], which was prepared from commercially available (*S*)-6-methyloctanol, was labeled with (*R*)-2A1P or (*S*)-2A1P to give reference samples, (*S*)-**5**-(*R*)-2A1P and (*S*)-**5**-(*S*)-2A1P (Supplementary Figures S7 and S8). Retention times of reference samples were 130.5 min for (*S*)-**5**-(*S*)-2A1P (corresponding to (*R*)-**5**-(*R*)-2A1P diastereomer) and 139.3 min for (*S*)-**5**-(*R*)-2A1P (Figure 3a). Surprisingly, *nat*-**5**-(*R*)-2A1P derived from **4** showed two peaks for (*R*)-**5**-(*R*)-2A1P and (*S*)-**5**-(*R*)-2A1P in a ratio of 2:3 (Figure 3b). Consequently, nocapyrone L (**4**) was confirmed as an enantiomeric mixture of (*R*)- and (*S*)-isomers in a ratio of 2:3. This was supported by the ^1^H NMR signal for C-6 methyl protons of *nat*-**5**-(*R*)-2A1P, which showed overlapped signals for those of (*S*)-**5**-(*S*)-2A1P and (*S*)-**5**-(*R*)-2A1P (Figure 4).

### 2.2. Biological Activities

**Figure 5:**
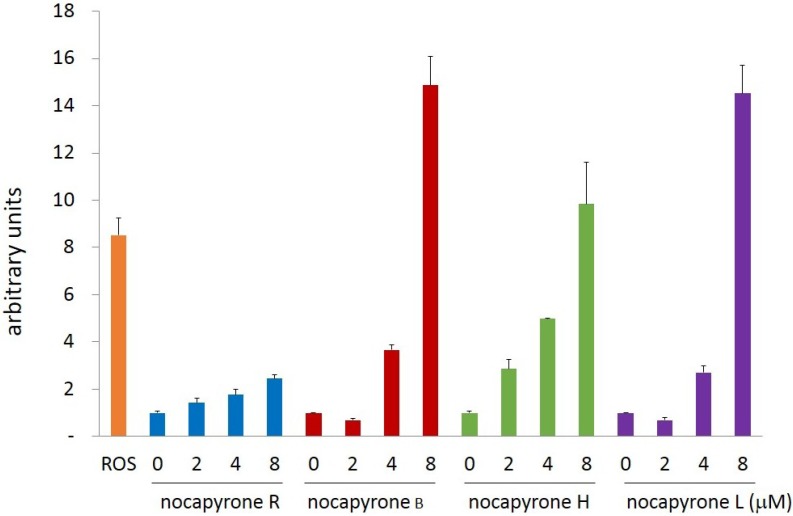
Effect of nocapyrones on expression of adiponectin mRNA (ROS = rosiglitazone, 20 nM).

**Figure 6:**
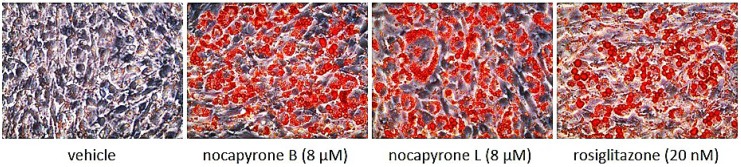
Adipocyte differentiation induced by nocapyrones B and L.

**Figure 7:**
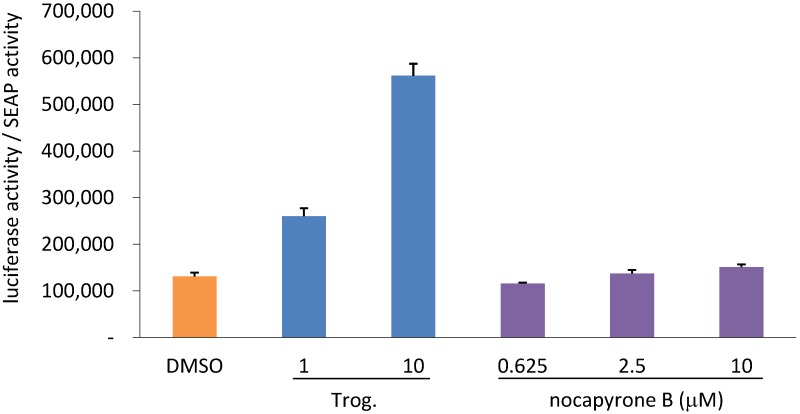
Effect of nocapyrone B on activation of PPARγ in luciferase ligand assay system.

Activity of compounds **1**–**4** to promote adiponectin-production in adipocyte cells was assessed by measuring the mRNA expression level of adiponectin-coding gene [25]. After the treatment of murine ST-13 preadipocyte cells with the compounds for 11 days, the total mRNA was subjected to quantitative real-time PCR analysis. All the γ-pyrones (**2**–**4**) enhanced the expression of adiponectin mRNA in a concentration-dependent manner while the α-pyrone (**1**) showed no such effect (Figure 5). Especially, **2** and **4** more strongly induced adiponectin production at 8 μM and at the same concentration the accumulation of lipid droplets were observed (Figure 6, oil red O staining) that indicates the differentiation to mature adipocyte cells. Compound **2** was further examined for the transcriptional activation of PPARγ that is the main target of the antidiabetic drug, thiazolidinediones. In the luciferase reporter assay, troglitazone activated the transcription through PPARγ but **2** did not (Figure 7), confirming that **2** is not a ligand for PPARγ and its mode of action is different from thiazolidinediones. These effects on adipocytes or adiponectin-inducing activity have not been described for nocapyrones and other related pyrone compounds.

## 3. Experimental Section

### 3.1. General Experimental Procedures

Optical rotation was measured using a JASCO DIP-3000 polarimeter (JASCO Corporation, Hachioji, Japan). UV spectra were recorded on a Hitachi U-3210 spectrophotometer (Hitachi High-Technologies Corporation, Tokyo, Japan). IR spectra were measured on a Perkin-Elmer Spectrum 100 (Perkin-Elmer Japan Co. Ltd., Yokohama, Japan). NMR spectra were obtained on a Bruker AVANCE 400 (Bruker BioSpin K.K., Yokohama, Japan) or a Bruker AVANCE 500 spectrometer (Bruker BioSpin K.K., Yokohama, Japan). HR-ESI-TOFMS were recorded on a Bruker microTOF focus (Bruker Daltonics K.K., Yokohama, Japan). Cosmosil 75C18-PREP (Nakalai Tesque, Inc., Kyoto, Japan, 75 μm) was used for ODS column chromatography.

### 3.2. Microorganism

Strain TP-A0876 was isolated from a sediment sample collected at a depth of 775 m off the island of Hokkaido (N 42°56′80″, E 140°06′32″), Japan in 2006 (August), using a piston corer. The strain was identified as a member of the genus *Nocardiopsis* on the basis of 100% 16S rRNA gene sequence (1455 nucleotides; GenBank accession number AB488799) similarity to *Nocardiopsis* sp. 123 (accession number AY036002).

### 3.3. Fermentation

Strain TP-A0876 cultured on a Bn-2 agar plate soluble starch 0.5%, glucose 0.5%, meat extract (Kyokuto Pharmaceutical Industrial Co., Ltd., Tokyo, Japan) 0.1%, yeast extract (Difco Laboratories, Surrey, United Kingdom) 0.1%, NZ-case (Wako Pure Chemical Industries, Ltd., Osaka, Japan) 0.2%, NaCl 0.2%, CaCO_3_ 0.1% and agar 1.5%] was inoculated into 500-mL K-1 flasks each containing 100 mL of the V-22 seed medium consisting of soluble starch 1%, glucose 0.5%, NZ-case 0.3%, yeast extract 0.2%, Tryptone (Difco Laboratories) 0.5%, K_2_HPO_4_ 0.1%, MgSO_4_·7H_2_O 0.05%, and CaCO_3_ 0.3% (pH 7.0). The flasks were placed on a rotary shaker (200 rpm) at 30 °C for 4 days. The seed culture (3 mL) was transferred into 500-mL K-1 flasks each containing 100 mL of the A-3M production medium consisting of glucose 0.5%, soluble starch 2.0%, glycerol 2.0%, yeast extract 0.3%, Pharmamedia (Trader’s Protein) 1.5%, and Diaion HP-20 (Mitsubishi Chemical Holdings Corporation, Tokyo, Japan) 1%. The pH of the medium was adjusted to 7.0 before sterilization. The inoculated flasks were placed on a rotary shaker (200 rpm) at 30 °C for 7 days.

### 3.4. Extraction and Isolation

At the end of the fermentation period, 100 mL of 1-butanol was added to each flask, and they were allowed to shake for one hour. The mixture was centrifuged at 5000 rpm for 10 min and the organic layer was separated from the aqueous layer containing the mycelium. Evaporation of the solvent gave 7.8 g of extract from 2 L of culture. The crude extract (7.8 g) was subjected to silica gel column chromatography with a step gradient of CHCl_3_/MeOH (1:0, 20:1, 10:1, 4:1, 2:1, 1:1, and 0:1 *v/v*). Fractions 2 (20:1) and 3 (10:1) were combined and concentrated *in vacuo* to give brown oil (0.97 g) which was further fractionated by reverse-phase ODS column chromatography with a gradient of MeCN/distilled water (2:8, 3:7, 4:6, 5:5, 6:4, 7:3, and 8:2 *v/v*). Fractions 4 (5:5) and 5 (6:4) were combined and evaporated and the remaining aqueous solution was extracted with EtOAc. The organic layer was washed with brine, dried over anhydrous Na_2_SO_4_, filtered, and evaporated to dryness. The residual brown solid (0.28 g) was then subjected to HPLC purification using an ODS column (Cosmosil ARII, 10 mm × 250 mm) with 55% MeCN in distilled water at 3.0 mL/min to give nocapyrone B (**2**, *t*_R_ 28.1 min, 10 mg), nocapyrone H (**3**, *t*_R_ 31.3 min, 2.3 mg) and a mixture of nocapyrones R and L (**1** and **4**, *t*_R_ 41.2 min, 3.8 mg). **1** and **4** were separated by using a cholesterol-bonded reverse-phase column (Cosmosil Cholester, 10 mm × 250 mm) with 55% MeCN in distilled water at 3.0 mL/min to give **4** (*t*_R_ 29.9 min, 2.1 mg) and **1** (*t*_R_ 35.0 min, 0.8 mg).

Nocapyrone R (**1**): colorless amorphous solid; UV (MeOH) λ_max_ (log ε) 290 (4.04) nm; IR (ATR) ν_max_ 2944, 1660 cm^−1^; ^1^H and ^13^C-NMR data, see Table 1; HRESITOFMS *m*/*z* 275.1619 [M + Na]^+^ (calcd for C_15_H_24_O_3_Na, 275.1623).

### 3.5. Determination of the Absolute Configuration of the Anteiso-Methyl Group in 4 by Ohrui-Akasaka Method

Oxidative Degradation of **4** to Yield **5**: A solution of nocapyrone L (**4**, 4 mg, 15 μmol) in a mixture of MeCN (320 μL) and H_2_O (240 μL) was stirred with NaIO_4_ (19.2 mg, 89 μmol) until the salt was dissolved. To this solution were added CCl_4_ (320 μL) and 1 mg/mL solution of RuCl_3_ hydrate in 0.1 M sodium phosphate buffer (240 μL, pH 7.6) and the biphasic mixture was vigorously stirred at room temperature for 17 h. The reaction mixture was passed through Celite and the filter cake was washed with MeCN. After evaporation of the organic solvent from the filtrate, the aqueous solution was acidified with 2 M HCl and extracted with EtOAc. The EtOAc layer was concentrated and the remaining material was dissolved in MeOH (1 mL) and THF (1 mL). To this solution was added 1 M NaOH (1 mL) and the mixture was stirred at room temperature for 12 h. The reaction mixture was then acidified with 2 M HCl and extracted with EtOAc. The EtOAc layer was washed with water and brine, dried over anhydrous Na_2_SO_4_, and concentrated *in vacuo* to give 6-methyloctanoic acid (**5**, 1.2 mg) which was used for the next reaction without further purification:^1^H NMR (CDCl_3_, 500 MHz) δ 0.84 (3H, d, *J* = 6.0 Hz), 0.85 (3H, t, *J* = 7.6 Hz), 1.12 (2H, m), 1.28–1.35 (5H, m), 1.62 (2H, m), 2.36 (1H, t, *J* = 7.5 Hz).

Preparation of *nat*-**5**-(*R*)-2A1P: 6-Methyloctanoic acid (**5**) obtained by degradation of nocapyrone L (**4**) was reacted with (*R*)-2-(anthracene-2,3-dicarboximido)propanol [(*R*)-2A1P] (2 mg, 6.6 μmol) in dry CH_2_Cl_2_ (1 mL) in the presence of EDAC (2 mg, 10 μmol) and DMAP (trace amount) at room temperature for 17 h. The reaction mixture was diluted with ice-water and extracted with EtOAc. The organic layer was concentrated under reduced pressure and the residue was chromatographed over a silica gel column (*n*-hexane–EtOAc = 1:0~1:1) to give (*R*)-2A1P ester derivative of naturally occurring 6-methyloctanoic acid [*nat*-**5**-(*R*)-2A1P, 0.8 mg].

Preparation of standard (*S*)-6-methyloctanoic acid: To a solution of (*S*)-6-methyl-1-octanol (10 mg, 69 μmol, Wako Pure Chemical Industries, Ltd., Osaka, Japan) in acetone (1 mL) was added Jones reagent (0.4 mL, 80 μmol) dropwise. After stirring for 3 h at room temperature, the reaction mixture was extracted with EtOAc and the organic layer was concentrated *in vacuo* to give (*S*)-6-methyloctanoic acid (7.0 mg): ^1^H NMR (CDCl_3_, 500 MHz) δ 0.85 (3H, t, *J* = 5.5 Hz), 0.87 (3H, d, *J* = 7.0 Hz), 1.25–1.35 (5H, m), 1.33 (2H, m), 1.62 (2H, m), 2.36 (3H, t, *J* = 7.5 Hz).

Preparation of standard (*S*)-**5**-(*R*)-2A1P and (*S*)-**5**-(*S*)-2A1P: In the same manner as described for the preparation of *nat*-**5**-(*R*)-2A1P, (*S*)-**5**-(*R*)-2A1P and (*S*)-**5**-(*S*)-2A1P were prepared by the reaction of (*S*)-6-methyloctanoic acid with (*R*)- and (*S*)-2A1P, respectively.

(*S*)-**5**-(*R*)-2A1P: ^1^H NMR (CDCl_3_, 500 MHz) δ 0.72 (3H, d, *J* = 5.2 Hz), 0.74 (3H, t, *J* = 6.7 Hz), 0.97 (2H, m), 1.11–1.25 (5H, m), 1.49 (2H, m), 1.57 (2H, d, *J* = 7.1 Hz), 2.22 (2H, t, *J* = 7.7 Hz), 4.61 (1H, t, *J* = 6.1 Hz), 4.43 and 4.73 (2H, m), 7.62 (1H, dd, *J* = 6.5, 2.9 Hz), 8.07 (1H, dd, *J* = 6.3, 2.8 Hz), 8.48 (1H, s), 8.60 (1H, s).

(*S*)-**5**-(*S*)-2A1P: ^1^H NMR (CDCl_3_, 500 MHz) δ 0.71 (3H, d, *J* = 5.9 Hz), 0.74 (3H, t, *J* = 6.8 Hz), 0.96 (2H, m), 1.13–1.25 (5H, m), 1.48 (2H, m), 1.57 (2H, d, *J* = 7.1 Hz), 2.23 (2H, t, *J* = 7.7 Hz), 4.62 (1H, t, *J* = 6.1 Hz), 4.44 and 4.74 (2H, m), 7.62 (1H, dd, *J* = 7.0, 3.1 Hz), 8.07 (1H, dd, *J* = 6.3, 2.8 Hz), 8.48 (1H, s), 8.60 (1H, s).

HPLC analysis: *nat*-**5**-(*R*)-2A1P derived from nocapyrone L (**4**) and synthetic (*S*)-**5**-(*R*)-2A1P and (*S*)-**5**-(*S*)-2A1P were analyzed by HPLC under the following conditions. Column: Develosil ODS-HG-3 (4.6 mm × 150 mm, Nomura Chemical); mobile phase: MeOH/MeCN/THF=4:3:1; column temperature: −58.5 °C; flow rate: 0.2 mL/min. The column was cooled by using Cryocool CC-100 (Neslab Instruments Inc., Portsmouth, NH, USA). HPLC peaks were detected by monitoring fluorescence intensity at 460 nm with the excitation at 362 nm by using an FP-920 fluorescence detector (JASCO Corporation, Hachioji, Japan). Retention times were 130.5 min for (*S*)-**5**-(*S*)-2A1P and 139.3 min for (*S*)-**5**-(*R*)-2A1P. Natural product-derived *nat*-**5**-(*R*)-2A1P gave peaks at 130.8 min and 139.3 min in a ratio of 2:3.

### 3.6. Biological Activity Study

Adipocyte differentiation assay was carried out according to the procedure previously described [25].

#### 3.6.1. Real-Time Quantitative PCR Analysis

The total RNA (1 μg) was reverse-transcribed to cDNA using a Super Script TM II RT (Invitrogen, Tokyo, Japan). To quantitatively estimate the mRNA levels of several genes, PCR amplification was performed using a Light Cycler system (Roche Diagnostic Co., Tokyo, Japan). Real-time PCR was carried out in a total volume of 20 μL containing 500 nM each of gene-specific primers, cDNA, and SYBRP remix Ex Tag (Takara, Kyoto, Japan). Expression was normalized to glyceraldehyde-3-phosphate dehydrogenase (GAPDH). Thermal cycling conditions for the PCR were 95 °C for 5 min, followed by 45 cycles of 95 °C for 5 s, 60 °C for 15 s, and 72 °C for 10 s, then a melting curve analysis from 65 °C to 95 °C, every 0.1 °C. The primer sequences used were: adiponectin, 5′-GAAGCCGCTTATATGTATCG-3′ (forward) and 5′-GCCGTCATAATGATTCTGT-3′ (reverse); GAPDH, 5′-CCAGAACATCATCCCTGC-3′ (forward) and 5′-CCACGACGGACACATT-3′ (reverse); fatty acid-binding protein (aP2), 5′-GAAATCACCGCAGACG-3′ (forward) and 5′-ACATTCCACCACCAGC-3′ (reverse); PPARγ2, 5′-CTGTTGACCCAGAGCA-3′ (forward) and 5′-GCGAGTGGTCTTCCAT-3′ (reverse).

#### 3.6.2. Oil Red O Staining

ST-13 cells treated with nocapyrones B and L for 11 days were washed three times with phosphate-buffered saline (PBS), fixed with 10% formalin neutral buffer solution (Wako Pure Chemical, Osaka, Japan) at room temperature for 10 min, and then washed with distilled water to remove formalin solution. Furthermore, the cells were rinsed with 60% 2-propanol for 5 min, stained with 0.24% oil red O at room temperature for 20 min, and then were photographed under a phase contrast microscope (×100 magnification) equipped with a CCD camera (Leica Microsystems Japan, Tokyo, Japan).

#### 3.6.3. Luciferase Reporter Assay

An expression plasmid containing the ligand binding domain of human PPARγ fused to the GAL4 DNA binding domain (pPPARγ-GAL4), and the luciferase reporter plasmid, 17m2G TATA Luc (p17m2G), were kindly donated by Dr. S. Kato (University of Tokyo, Tokyo, Japan). We transiently transfected COS-1 cells (6 × 10^5^ cells) with pPPARγ-GAL4 (0.25 μg) and p17m2G (1 μg) using Effectene Transfection Reagent (QIAGEN, Tokyo, Japan). Transfections were performed in triplicate in 24-well plates according to the manufacture’s instructions. After 16 h, the transfected cells received the indicated concentrations of nocapyrones B (or troglitazone), and were cultured for additional 24 h at 37 °C in a 5% CO_2_ incubator. Then cells were harvested and luciferase activities were measured by a Steady-Glo^®^ Luciferase Assay System (Promega, Madison, WI, USA) according to manufacturer’s instructions.

## 4. Conclusions

In this study, a marine-derived actinomycete *Nocardiopsis* sp. TP-A0876 was isolated from a sediment sample and its secondary metabolites were investigated. Purification from the 1-butanol extract of the culture broth resulted in isolation of one α-pyrone and three γ-pyrones. Based on the spectroscopic analysis, the α-pyrone was confirmed as a new compound, whereas other three γ-pyrones were identified as nocapyrones B (**2**), H (**3**), and L (**4**). Biological testing in a set of assays proved that γ-pyrone compounds (**2**–**4**) have ability to induce preadipocyte differentiation and adiponectin production in murine ST13 preadipocyte cells. Since the α-pyrone compound **1** showed no activity, the γ-pyrone structure is proposed to play a key role in this activity.

**Figure 8:**
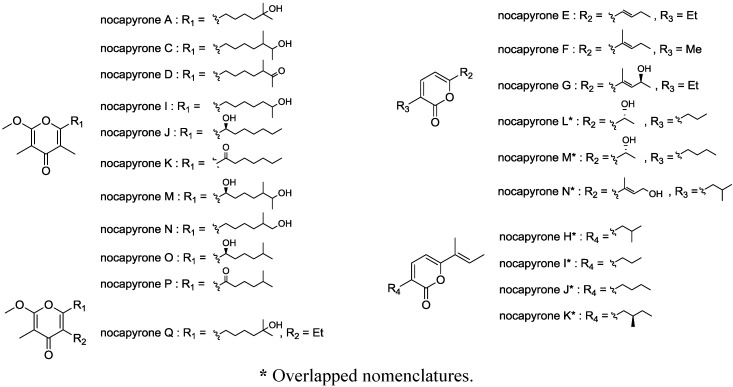
Nocapyrones isolated from marine-derived *Nocardiopsis*.

A large number of pyrone–containing marine natural products are known [26,27,28,29,30,31,32]. Specifically, a series of nocapyrones (A–Q) have been continuously reported from marine-derived *Nocardiopsis* strains with a wide range of bioactivity (Figure 8). Nocapyrones B (**2**) and H (**3**) modulate nerve cell depolarization, activate or inhibit Ca^2+^ flux into a panel of human cells overexpressing various transient receptor potential (TRP) channels depending upon the agent and channel subtype, together with cytotoxicity against human breast adenocarcinoma, but have no antibacterial activity [21]. Nocapyrones E–G are described to have modest antibacterial activity against *Bacillus subtilis* [30]. Nocapyrone H* inhibits NO production in LPS-stimulated BV-2 microglial cells and neuro-protective effect [31], and nocapyrones H*, I*, and M* were shown to suppress gene expression in quorum sensing [32]. However, there is no report on the activity of these pyrones for preadipocyte differentiation or adiponectin production. Although the effectiveness is lower than thiazolidinediones, different mode of action of the γ-pyrones may provide an opportunity of drug development from this new therapeutic scaffold for insulin resistance type 2 diabetes. The concise synthetic strategies [33,34,35,36] and interesting bioactivities of structurally related compounds have been reported [37,38]. On the base of these studies, further synthetic and biological studies of nocapyrone families could be inspiring.

**Figure 9:**
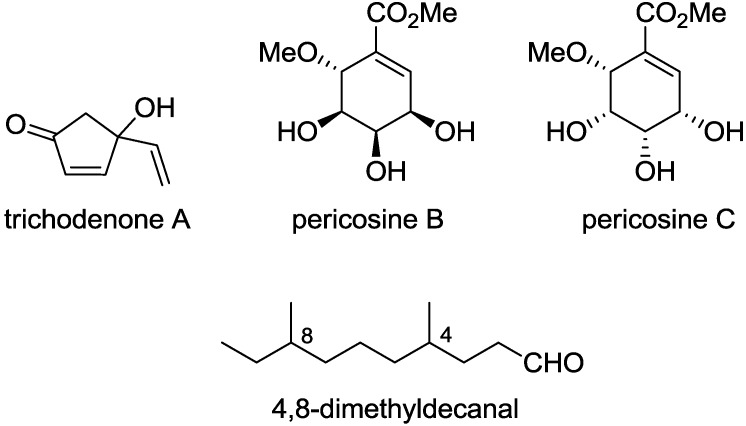
Natural products existing as mixtures of enantiomers.

The absolute configuration of *anteiso*-methyl group is known and believed to be *S* in most cases because it is derived from l-isoleucine. However, the *anteiso*-methyl branching in **4** was confirmed as a mixture of (*R*)- and (*S*)-configurations in a ratio of 2:3. Along with the advances in synthetic and analytical techniques, several enantiomeric mixtures have been discovered in natural products (Figure 9) [39,40,41]. Many of them are marine-derived: trichodenone A, an antitumor metabolite of *Trichoderma harzianum* isolated from marine sponge (*Halichondria okadai*), is an (*R*)- and (*S*)-enantiomeric mixture [39]; pericosines B and C, metabolites of *Periconia byssoide* isolated from sea hare *Aplysia kurodai*, bearing four chiral centers are also enantiomeric mixtures [40]. Recently, the enantiomeric mixture regarding to the *anteiso*-methyl asymmetry was found in the insect pheromone. 4,8-Dimethyldecanal, the male aggregation pheromone of the red flour beetle (*Tribolium castaneum*), consists of four diastereomers, (4*R*, 8*R*)-, (4*R*, 8*S*)-, (4*S*, 8*R*)-, and (4*S*, 8*S*)-forms in a ratio of 4:4:1:1 [41]. To the best of our knowledge, nocapyrone L (**4**) is the first microbial metabolite proved to comprise an enantiomeric pair of *R*- and *S*-configurations at the *anteiso*-methyl substituent. The *sec*-methyl group at C-10 of nocapyrones C [20] and M [21] are reported to be an *R*/*S*-mixture but these compounds have a hydroxyl group on the neighboring carbon [21]. Biosynthetic origin of their *anteiso*-methyl portion should be clarified. Further investigation, focused on the biosynthetic precursors and gene analysis with our nocapyrone H (**4**), are under way to identify the enzymatic reactions responsible for the formation of enantiomeric mixture [42].

* Overlapped nomenclatures.

## Supplementary Files

Supplementary File 1 Supplementary Information (PDF, 477 KB)
