# Supplementary material for: Nocapyrones: α- and γ-Pyrones from a Marine-Derived Nocardiopsis sp
Source: Mar Drugs. 2014 Jul 8;12(7):4110–25. doi: 10.3390/md12074110 (PMC4113818; doi:10.3390/md12074110)
Supplement: Supplementary File 1 — Supplementary Information (PDF, 477 KB) [file marinedrugs-12-04110-s001.pdf]

Supplementary Information

Figure S1. ^{1}H NMR spectrum of nocapyrone R (**1**) at 500 MHz in $CDCl_3$.
Figure S2. ^{13}C NMR spectrum of nocapyrone R (**1**) at 100 MHz in $CDCl_3$.
Figure S3. ^{1}H-^{1}H COSY spectrum of nocapyrone R (**1**) at 500 MHz in $CDCl_3$.
Figure S4. HSQC spectrum of nocapyrone R (**1**) at 500 MHz in $CDCl_3$.
Figure S5. HMBC spectrum of nocapyrone R (**1**) at 500 MHz in $CDCl_3$.
Figure S6. ^{1}H NMR spectrum of *nat*-**5**-(*R*)-2A1P at 500 MHz in $CDCl_3$.
Figure S7. ^{1}H NMR spectrum of standard (*S*)-**5**-(*S*)-2A1P at 500 MHz in $CDCl_3$.
Figure S8. ^{1}H NMR spectrum of standard (*S*)-**5**-(*R*)-2A1P at 500 MHz in $CDCl_3$.

Figure S1. ^{1}H NMR spectrum of nocapyrone R (**1**) at 500 MHz in $CDCl_3$.

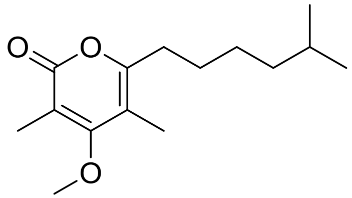

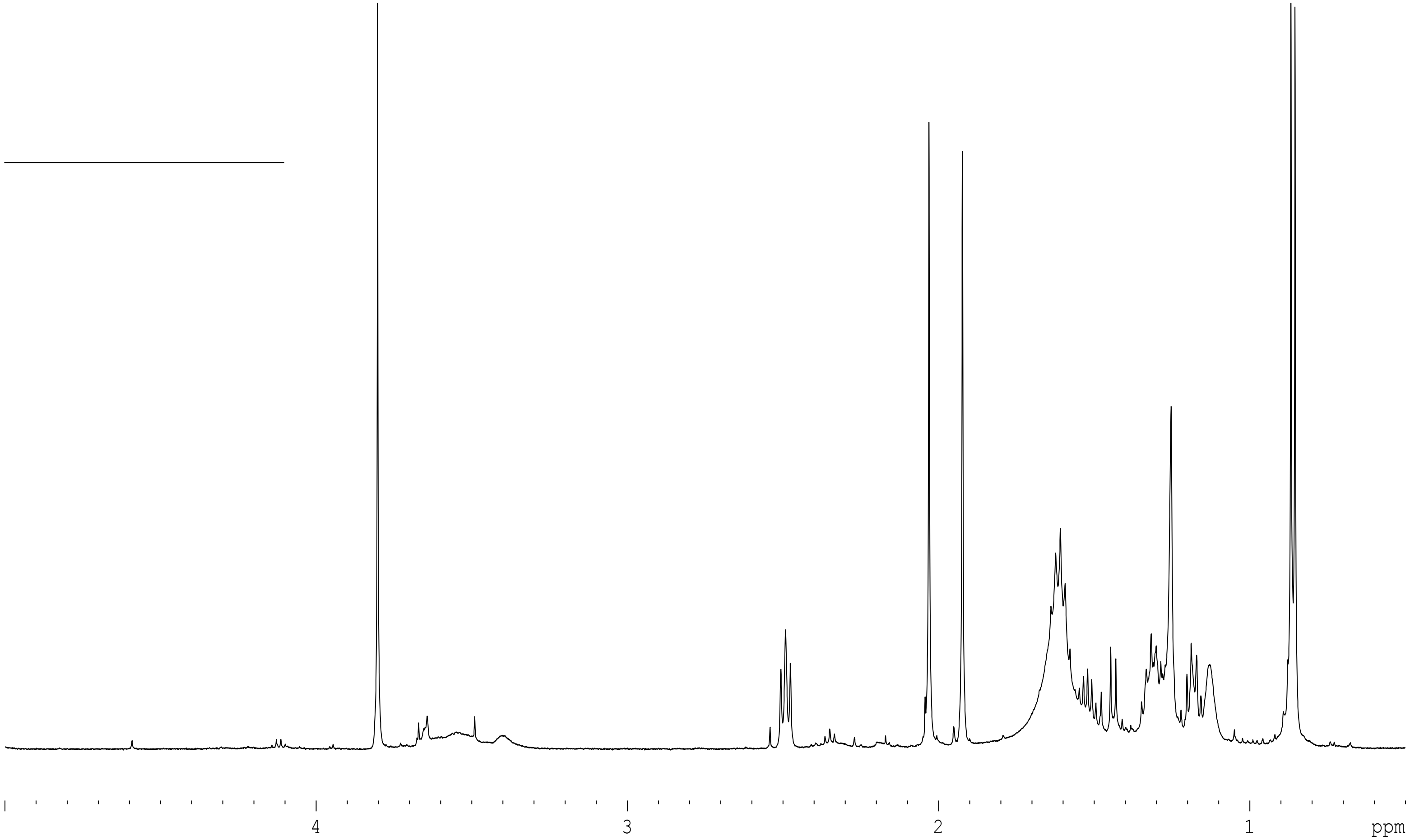

Figure S2. ^{13}C NMR spectrum of nocapyrone R (**1**) at 100 MHz in $CDCl_3$.

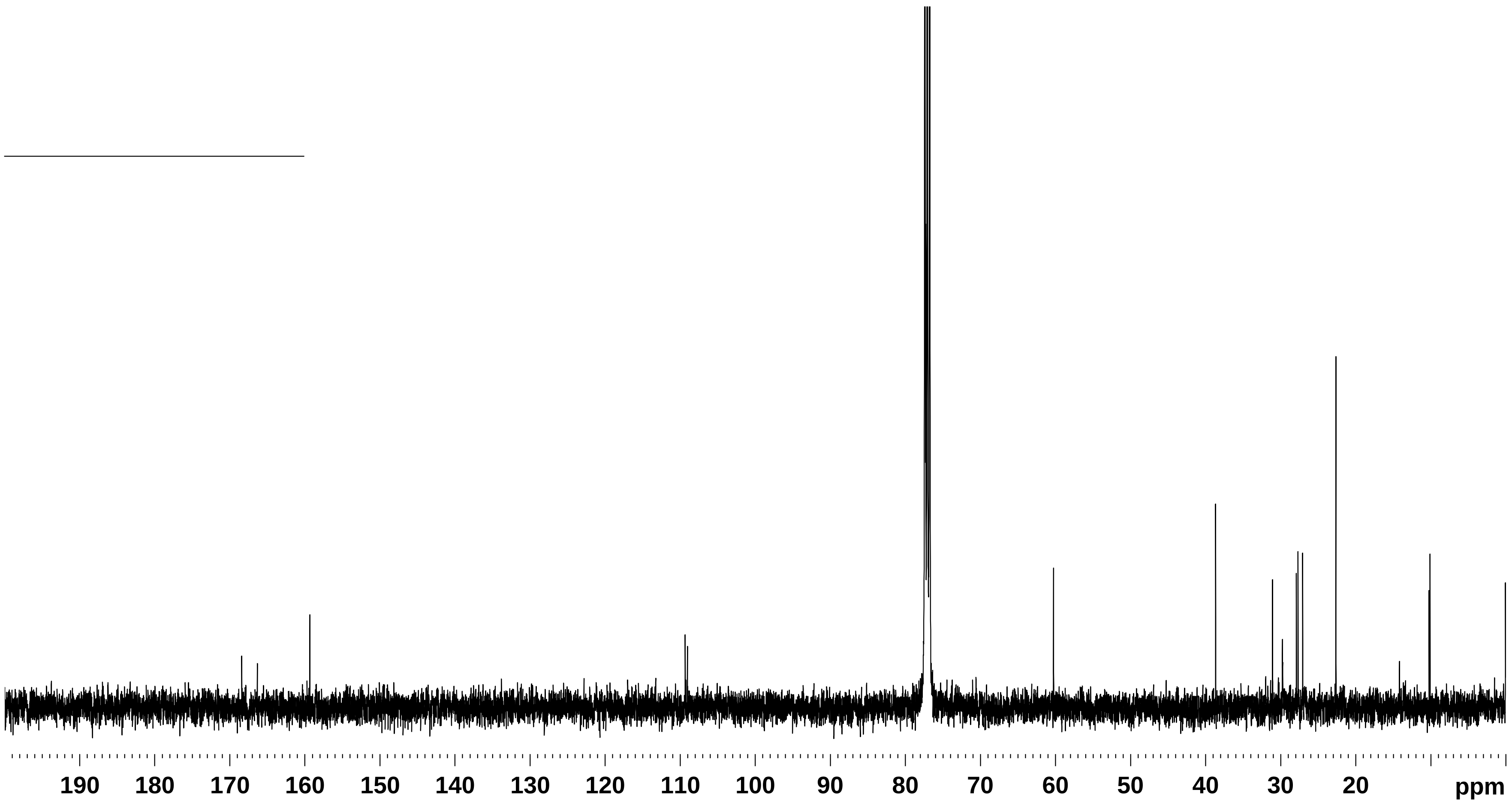

Figure S3. ^1H–^1H COSY spectrum of nocapyrone R (**1**) at 500 MHz in $CDCl_3$.

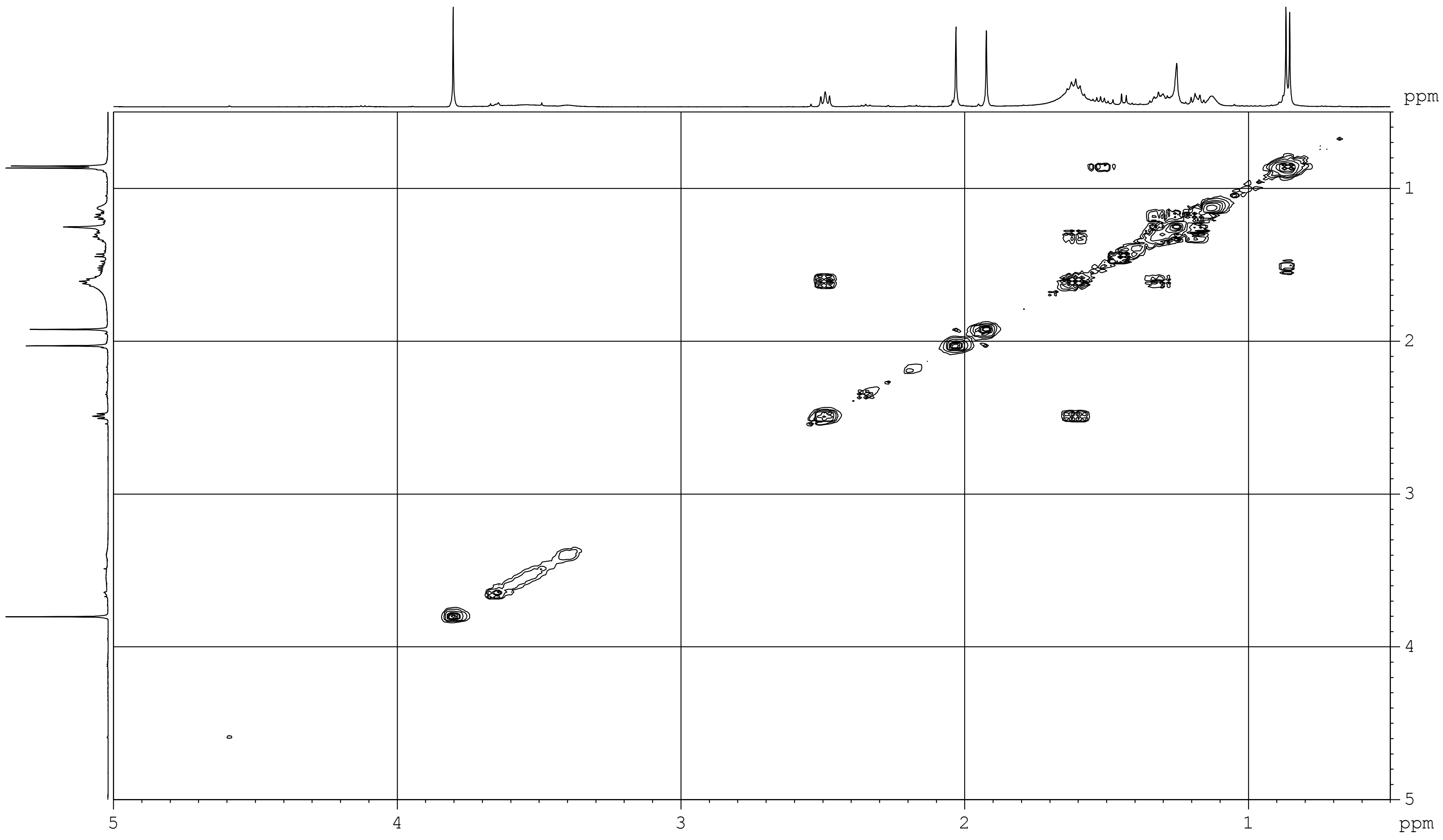

Figure S4. HSQC spectrum of nocapyrone R (**1**) at 500 MHz in $CDCl_3$.

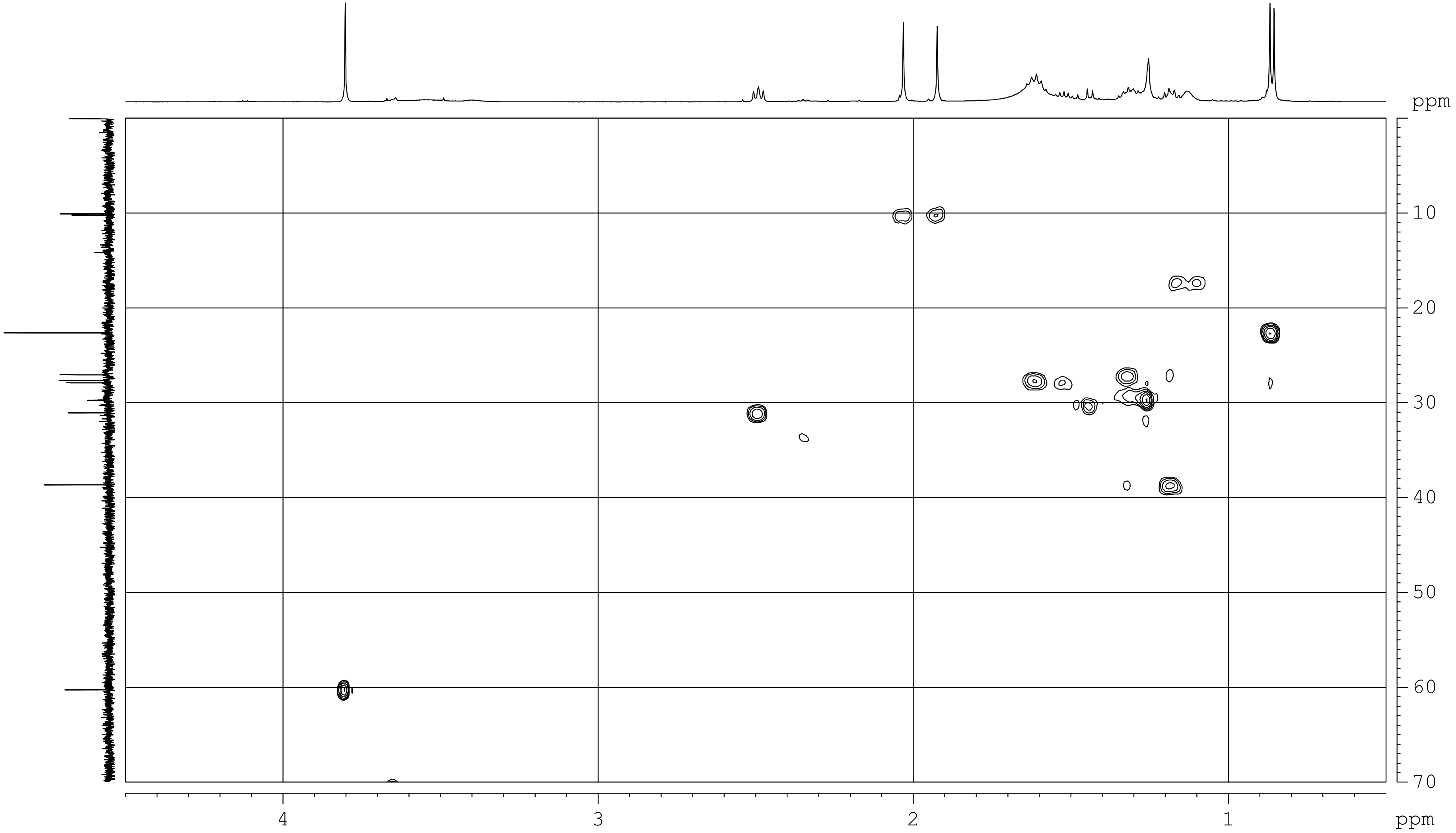

Figure S5. HMBC spectrum of nocapyrone R (**1**) at 500 MHz in $CDCl_3$.

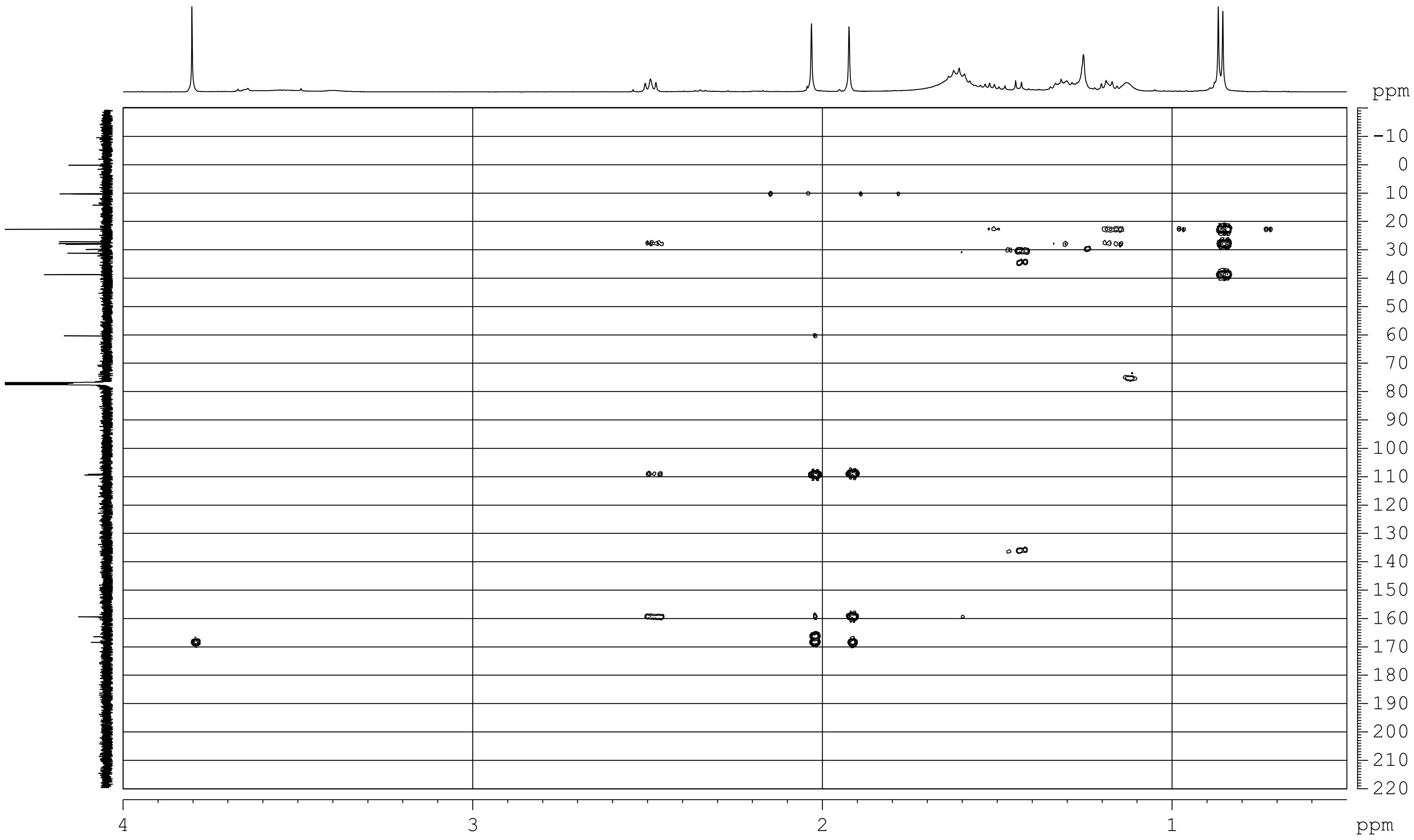

Figure S6. ^{1}H NMR spectrum of *nat*-**5**-(*R*)-2A1P at 500 MHz in $CDCl_3$.

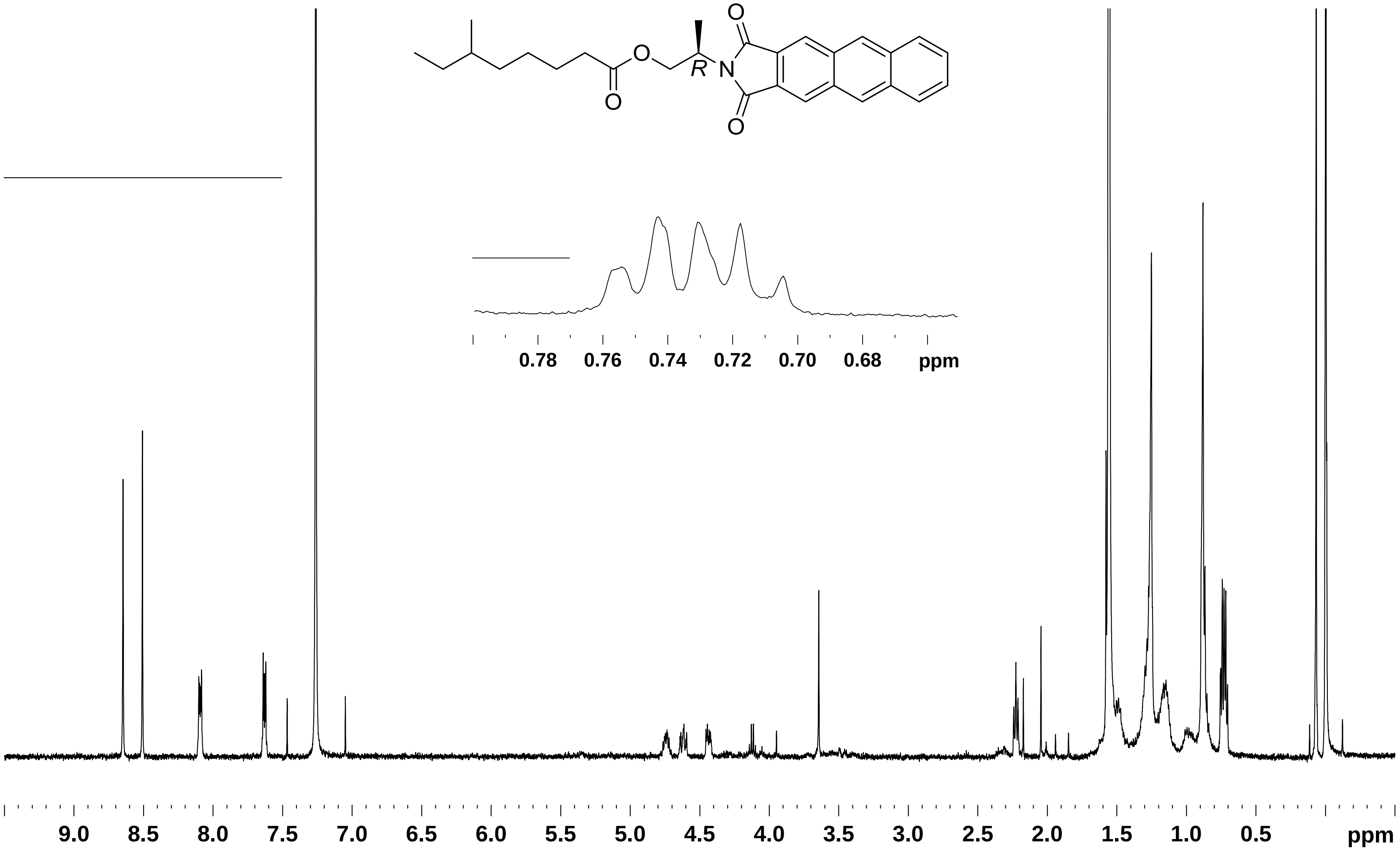

Figure S7. ^{1}H NMR spectrum of standard (*S*)-**5**-(*S*)-2A1P at 500 MHz in $CDCl_3$.

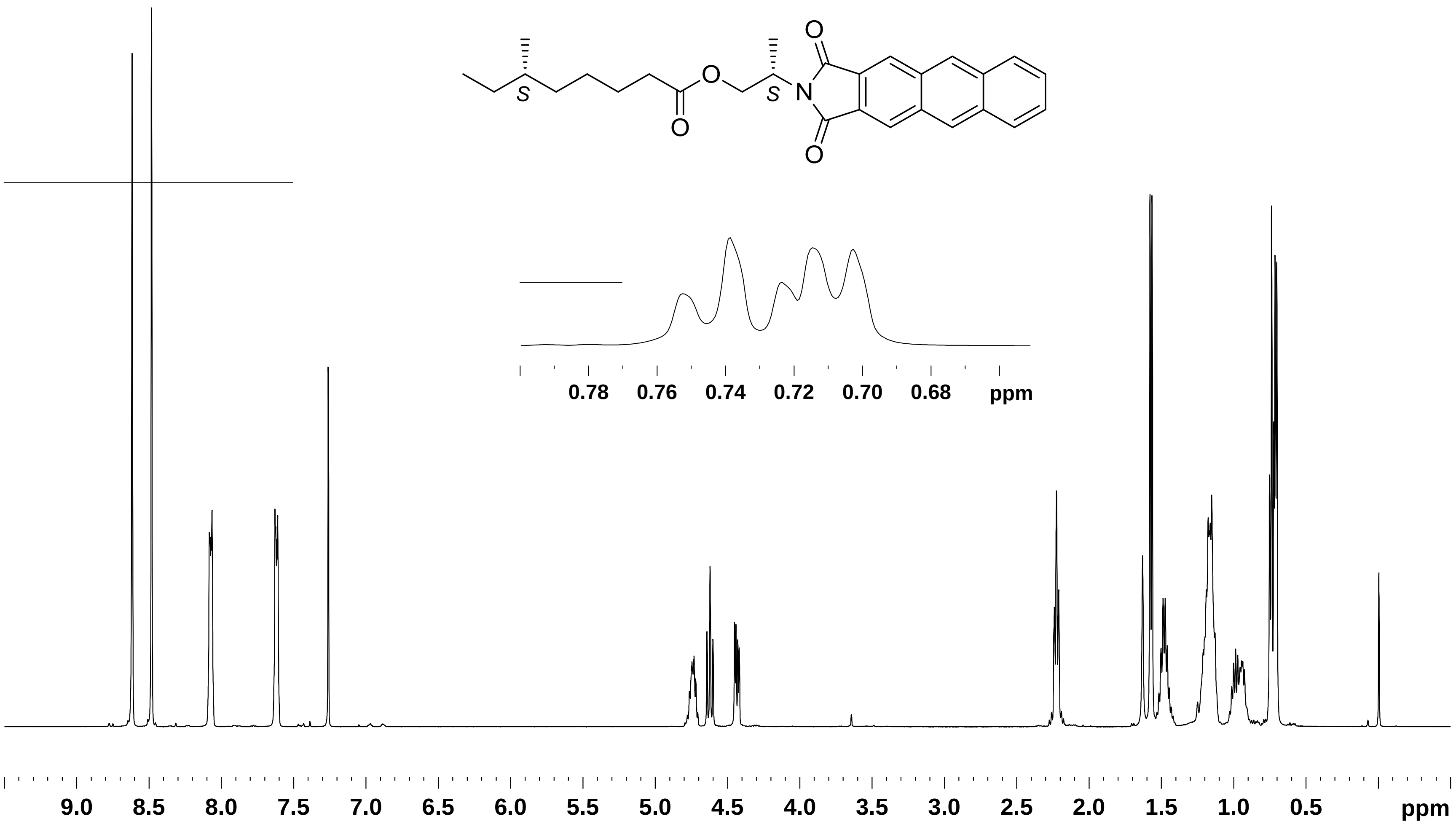

Figure S8. ^{1}H NMR spectrum of standard (*S*)-**5**-(*R*)-2A1P at 500 MHz in $CDCl_3$.

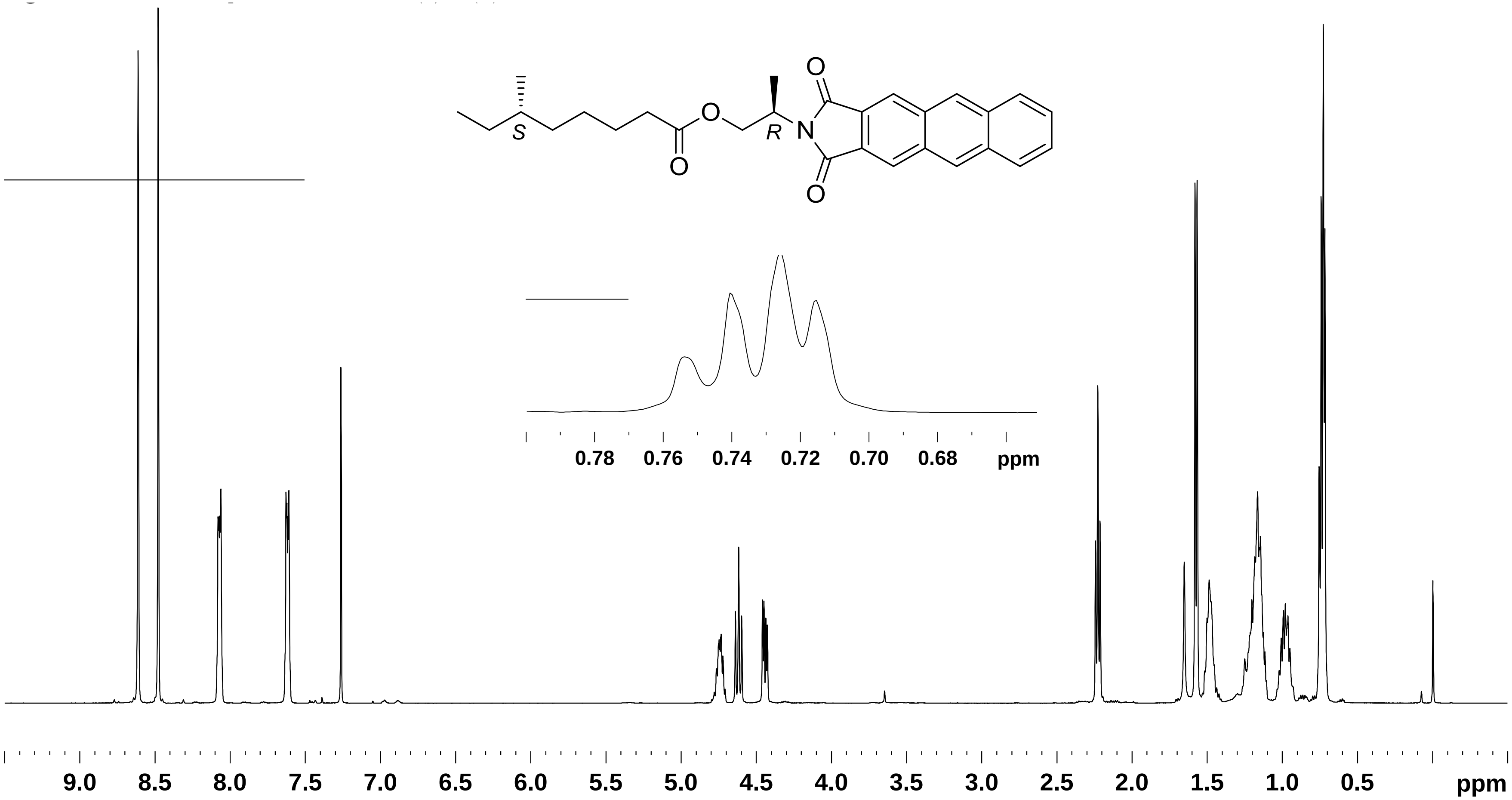

© 2014 by the authors; licensee MDPI, Basel, Switzerland. This article is an open access article distributed under the terms and conditions of the Creative Commons Attribution license (http://creativecommons.org/licenses/by/3.0/).
